# Draft genome sequence of carbapenem resistant *Escherichia coli* ST2083 carrying NDM-5, TEM-1, and CMY-42

**DOI:** 10.1128/mra.00519-24

**Published:** 2024-09-24

**Authors:** Rittwika Banerjee, Ramanakishore V. S., Vignesh S., Karthic G., Janani B., Marquess Raj, Kumar Perumal

**Affiliations:** 1 Department of Biotechnology, Sri Ramachandra Institute of Higher Education and Research, Chennai, Tamil Nadu, India; 2 Apollo Diagnostics, Regional Reference Laboratory, Chennai, Tamil Nadu, India; Loyola University Chicago, Chicago, Illinois, USA

**Keywords:** *Escherichia coli*, carbapenems, ST2083, NDM-5, CMY-42, TEM-1

## Abstract

The draft genome sequence of the *Escherichia coli* strain (CREC-9) of sequence type (ST2083), which was isolated from the urine sample of a 69-year-old male and harbors the antimicrobial resistance genes *TEM-1, blaCMY-42,* and *blaNDM-5* encoding resistance to β-lactams, cephalosporins, carbapenems, and fluoroquinolones and coding various virulence factors, is presented here.

## ANNOUNCEMENT

**Fig 1:**
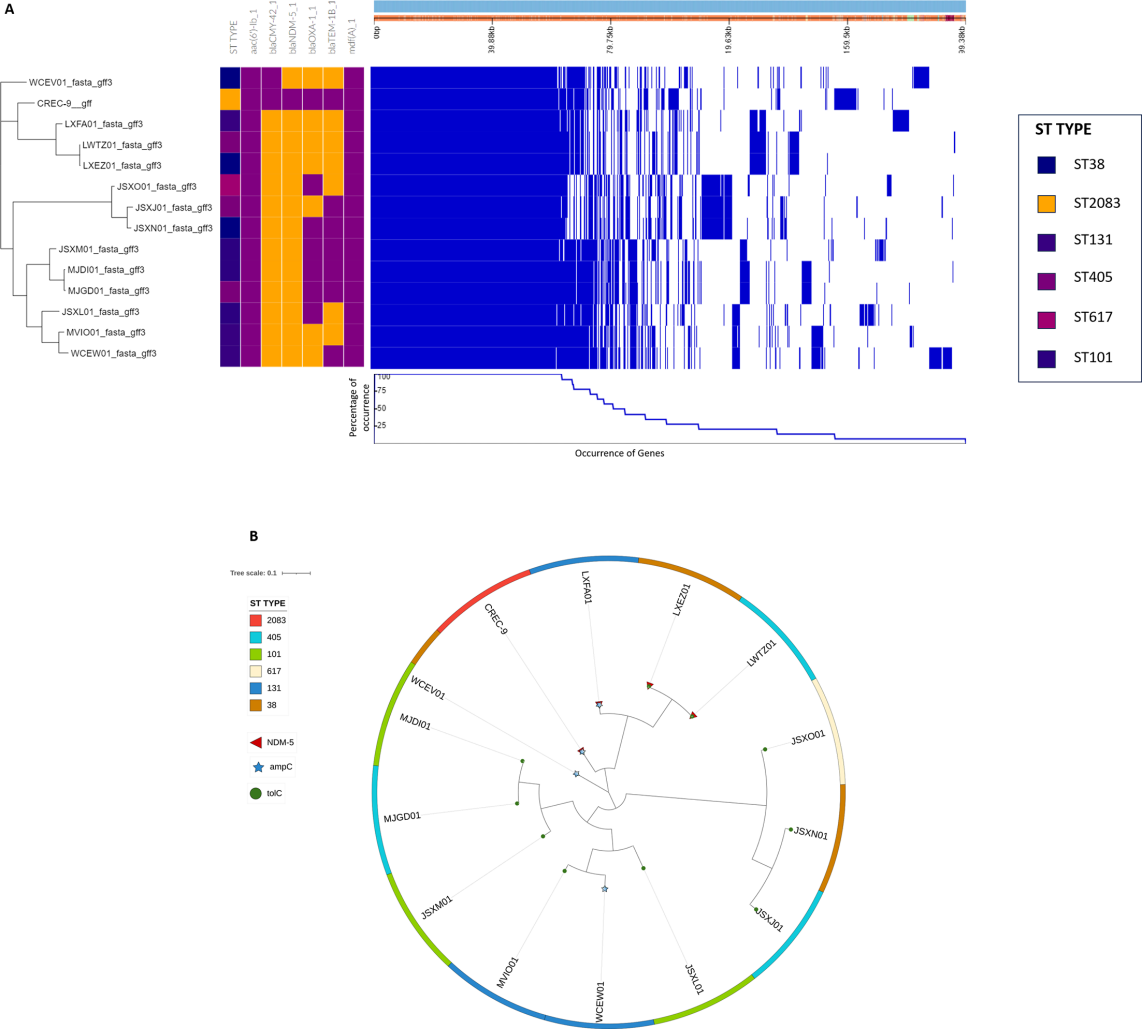
(A) Phandango visualization of 14 *Escherichia coli* isolates along with their six different ST types as mentioned along with their presence and absence with different color variations. The presence of AMR genes such as blaCMY-42, blaNDM-5, and mdf(A) indicated in purple. Meanwhile, the presence of blaOXA-1 is indicated in the orange-colored strip, and absence of *aac(6')-Ib* is seen in purple under respective gene columns. Blue color indicates the occurrence of genes (core genes, shell genes, and cloud genes) along with their percentage below in the entire region. (B) Phylogenetic tree construction for 14 *Escherichia coli* isolates of six different ST types (ST2083, ST405, ST101, ST617, ST131, and ST38) visualized using iTOL v6.9.1

One of the most frequent bacterial infections seen in urinary tract infections (UTIs) is caused by *Escherichia coli* (52.3%), according to the ICMR report 2022 (1) and World Health Organization (WHO) (2). Carbapenem-resistant *Escherichia coli* (CREC-9) was isolated from a 69-year-old male, collected from a diagnostic laboratory situated in Chennai, India, with the approval of the IEC for student projects at SRIHER (Approval Number-CSP/24/FEB/143/55). The bacterial isolate was collected on blood agar from the diagnostic laboratory and subcultured in MacConkey agar and grown for 24 hours at 37°C. It was further subcultured in EMB agar and nutrient agar under the same condition. *Escherichia coli* was identified using MALDI-TOF (3). A single colony was inoculated and cultured in Luria–Bertani broth for 18–24 hours at 37 ° C in an orbital shaker at 180 rpm. DNA isolation was done after 24 hours using the DNeasy Ultraclean Microbial Kit (4). The KAPA Hyperplus kit was used for library preparation. Whole-genome sequencing (WGS) was carried out using Illumina Novaseq 6000. The total raw reads were 18,495,072 with a read length of 150 bp for both reads (R1 and R2). Illumina adapter sequences were trimmed using Trim Galore (v0.6.7) (5) and assembled using Unicycler (v0.5.0) (6), containing 152 contigs providing a N50 value of 1,64,514 bp with a genome size of 5,032,363 bp and genome coverage of 551.28X. Quality control was checked using FASTQC (v0.12.1) (7). The sequence type was found to be ST2083 using pubMLST (8), and PATRIC (9) was used to identify virulence genes such as *acrB, allB, astA, csgB, entB, entC, entD,* and *entF*. The PGAP annotation (v5.2) (10) revealed the presence of plasmids like Col (BS512), Col (MG828), IncFIA, IncFIB, IncFII, and IncI. ST2083 was resistant to beta-lactams (ampicillin & amoxicillin–clavulanic acid), cephalosporins & carbapenems, carrying NDM-5, CMY-42, and TEM-1 AMR genes identified using AMRFinderPlus (v3.12.8) (11). In addition, genes like *aac(6')-Ib-cr* and *aac(6')-Ib-cr* were seen to be harboring fluoroquinolone and sul1 for sulfonamide resistance. CMY-42 and TEM-1 were seen conferring resistance against cephalosporins and beta-lactams along with NDM-5 conferring resistance to carbapenem and cephalosporin (12). Comparative pan-genome analysis was done using Roary (v3.13.0) (13) for 14 *Escherichia coli* ST types (India) obtained from the BacWGSTdb database (14). Default parameters were used for all softwares. A phylogenetic tree based on cgMLST (8) indicates relations between ST2083 and other ST types like ST405, ST131, ST101, and ST617. The Roary output was visualized using Phandango (v1.3.1) (15), revealing a significant amount of genes present in all 14 sequences of UTI patients from India, including CREC-9. It consists of core genes (31.66%), Shell genes (36.37%), and cloud genes (31.97%). Pan-genome analysis reveals the unique presence of NDM-5 in ST2083, conferring carbapenem resistance (16). Other ST types, such as ST131, ST405, ST38, and ST2083, show the presence of *marA* and *gadX* genes conferring fluoroquinolone and tetracycline resistance in all ST types (17) (Fig. 1A and B).

## Data Availability

The draft genome sequence of *Escherichia coli* ST2083 has been submitted at DDBJ/ENA/GenBank under BioProject identification No. PRJNA1098495 and BioSample identification No. SAMN40910138 with the accession No. JBBWUQ000000000. Raw sequence reads are uploaded at the NCBI SRA database SRR28681301.
